# Cerebral, subcortical, and cerebellar activation evoked by selective stimulation of muscle and cutaneous afferents: an fMRI study

**DOI:** 10.1002/phy2.270

**Published:** 2014-04-06

**Authors:** Daniel L. Wardman, Simon C. Gandevia, James G. Colebatch

**Affiliations:** 1 Faculty of Medicine, University of Sydney, Sydney, 2052, New South Wales, Australia; 2 Neuroscience Research Australia, Barker Street, RandwickSydney, 2031, New South Wales, Australia; 3 Prince of Wales Hospital Clinical School, University of New South Wales, Sydney, 2052, New South Wales, Australia

**Keywords:** Cerebellum, cortex, cutaneous afferents, evoked potentials, fMRI, movement disorders, muscle, Muscle afferents, sensorimotor

## Abstract

We compared the brain areas that showed significant flow changes induced by selective stimulation of muscle and cutaneous afferents using fMRI BOLD imaging. Afferents arising from the right hand were studied in eight volunteers with electrical stimulation of the digital nerve of the index finger and over the motor point of the FDI muscle. Both methods evoked areas of significant activation cortically, subcortically, and in the cerebellum. Selective muscle afferent stimulation caused significant activation in motor‐related areas. It also caused significantly greater activation within the contralateral precentral gyrus, insula, and within the ipsilateral cerebellum as well as greater areas of reduced blood flow when compared to the cutaneous stimuli. We demonstrated separate precentral and postcentral foci of excitation with muscle afferent stimulation. We conclude, contrary to the findings with evoked potentials, that muscle afferents evoke more widespread cortical, subcortical, and cerebellar activation than do cutaneous afferents. This emphasizes the importance, for studies of movement, of matching the kinematic aspects in order to avoid the results being confounded by alterations in muscle afferent activation. The findings are consistent with clinical observations of the movement consequences of sensory loss and may also be the basis for the contribution of disturbed sensorimotor processing to disorders of movement.

## Abstract (precis)

We studied the patterns of cerebral and cerebellar activation following selective activation of muscle and cutaneous afferents from the hand. Both modalities evoked cortical and cerebellar activations. Overall, areas of significant activation were more numerous for muscle afferent stimulation. Contrary to the findings with evoked potentials, we conclude that muscle afferents evoke more widespread cortical, subcortical, and cerebellar activation than do cutaneous afferents.

## Introduction

Both muscle and skin are richly innervated with specialized receptors which provide sensory information to the central nervous system. While many nerves are mixed, there exist both primary sensory and primary motor (including afferents) nerves. One‐third to one‐half of the myelinated fibers in mammalian muscle nerves are afferent (Sherrington 1894). Projections to the somatosensory cortex have been previously studied using direct electrical stimulation of peripheral nerves. Electrical stimulation of the median nerve depolarizes motor axons and large diameter, fast conducting sensory fibers of types A*α* and A*β* (Groups I and II). These derive from cutaneous receptors, muscle spindles (1° and 2°), Golgi tendon organs, and joint capsule receptors (Marani and Lakke 2012). Most imaging studies have concentrated on the cortical effects, particularly for the sensorimotor cortex. Electrical stimulation of the median nerve, or the application of air puff stimuli to the fingers, sequentially activates contralateral SI followed by posterior parietal cortex and bilateral SII, as measured with magnetoencephalography (Forss et al. 1994a,b). Functional MRI (1.5 T) has been used with electrical stimulation of the right median nerve, and has shown cortical activity in the SI hand area, with SII being activated at lower current values (Backes et al. 2000). Krause et al. (2001) in an fMRI (1.5 T) study showed that electrical stimuli applied to the fingers thus activating cutaneous nerve afferents only, could selectively activate areas 3b and the crown of the postcentral gyrus (area 1–2). It is also well known that muscle and skin afferent projections are not limited to the primary sensorimotor cortex. For example, passive joint movements, potentially activating both muscle and cutaneous afferents, have been shown to excite neurons within the motor cortex, thalamus, and basal ganglia (e.g., Lemon and Porter 1976; Horne and Porter 1980; Colebatch et al. 1990; Weiller et al. 1996; Lozano and Hutchison 2002), and SII (the secondary sensory area) is an additional projection area for both muscle and cutaneous afferents (Landgren et al. 1967). Joint afferents are thought to make only a minor contribution as they usually discharge only at the extremes of joint movement (McCloskey 1978).

The functional roles of cutaneous and muscle receptors are different. Low threshold cutaneous receptors for example, can provide information about the light touch of a caress, the texture of a surface and the presence and location of an insect crawling on the skin. Muscle receptors have a more direct role in motor control – for example in the production of a constant force contraction (Rothwell et al. 1982; Proske and Gandevia 2012). Both afferent species have a role in proprioception, particularly for the hand (Collins et al. 2000; for review Proske and Gandevia 2012). This important sense allows an individual to recognize the position and movement of the limbs and is required for the production of accurate and finely graded contractions. Our study was designed to compare the patterns of cortical activation evoked by a cutaneous versus a muscle afferent volley arising from the same region of the hand to examine whether these functional differences are reflected in the patterns of evoked supraspinal excitation.

## Methods

### Subjects

Eight male adult volunteers were recruited from the personnel employed at, or associates of, the then Prince of Wales Medical Research Institute (now Neuroscience Research Australia). The mean age (±SD) of the subjects was 40.4 ± 8.8 years (27–52 years) and the mean weight 79.3 ± 12.1 kg (55–95 kg). One subject was left‐handed. All subjects had no past medical history of neurological or psychiatric disease. All subjects signed a written statement of informed consent approved by the University of New South Wales Human Ethics Research Committee (HREC 03255).

### Stimulus paradigm

**Figure 1.**
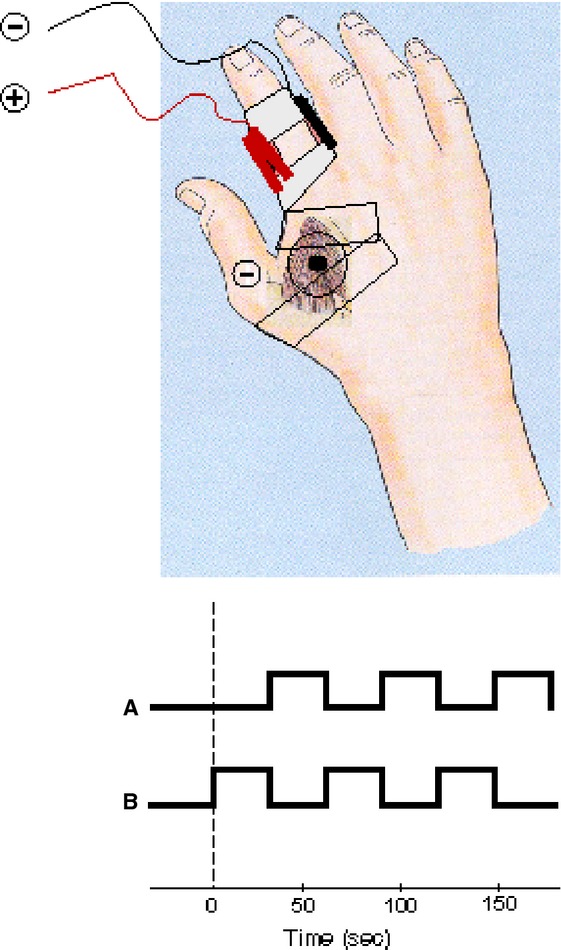
Experimental setup and stimulus block design. (Upper half) The cutaneous stimulus cathode was applied on the medial side of the index finger 1–2 cm distal to the metacarpophalangeal joint (black and red electrodes). The muscle stimulus cathode was applied over the motor point of the first dorsal interosseus muscle after delineation with a probe electrode (circle with black center). The stimulus anode in both cases was applied to the lateral skin (thumb side) of the right index finger approximately 1–2 cm distal to the metacarpophalangeal joint. (Lower half) The trial block design consisted of 8 fMRI collection runs. The order of the cutaneous or muscle stimulus blocks as well as the order of stimulus intensity (2T or 4T) were randomized. The different stimulus paradigms started with either a rest period followed by stimulation (A) or an initial stimulus (B) period (see text).

Electrical stimulation was applied via carbon electrodes (~3 cm^2^ Red Dot™ 3M, St Paul, MN) at two sites on the right hand. The anode for both cutaneous and muscle stimuli was applied to the lateral skin (thumb side) of the right index finger approximately 1–2 cm distal to the metacarpophalangeal joint (Fig. 1). The cutaneous stimulus cathode was applied on the medial side of the index finger 1–2 cm distal to the metacarpophalangeal joint. The muscle stimulus cathode was applied over the motor point of the first dorsal interosseous (FDI) muscle after delineation with a probe electrode. The skin over the motor point of FDI was anesthetized using a topical local anesthetic (EMLA^®^ AstraZeneca, Sydney, Australia: Lignocaine 25 mg/g and Prilocaine 25 mg/g) applied under nonporous occlusion (Tegaderm™ 3M, St Paul, MN) for 30–45 min prior to electrode placement to avoid direct cutaneous afferent stimulation. All electrodes were secured with adhesive tape (Micropore™ 3M, St Paul, MN). The electrical stimulus consisted of rectangular pulses at 4 Hz and 0.1 ms duration. Electrical stimulation of the finger at frequencies of 4–16 Hz produces good activation of the somatosensory cortex (Takanashi et al. 2003). Stimuli were generated by an isolated current stimulator (DS7 Digitimer Ltd, UK) located outside the room that was triggered by a purpose‐built timing circuit at ~3/sec. Electrical stimuli were delivered via 5‐m twisted copper cables connected to the carbon electrodes by clips that were also secured with adhesive tape (Fig. 1).

Individual subject thresholds for stimulus current were defined as the mean perceptual cutaneous threshold or the mean muscle twitch (motor) threshold. The mean cutaneous threshold was 7.3 ± 1.5 mA (range 5.5–10.0 mA) and the mean muscle stimulus threshold was 9.3 ± 2.5 mA (range 5.0–12.5 mA). Two different stimulus amplitudes were delivered in both the cutaneous and muscle conditions: twice (2T) and four (4T) times the stimulus threshold. Most subjects perceived the 4T muscle stimuli as somewhat painful.

The experimental design for each subject consisted of eight separate fMRI experimental runs as well as both T2 and 3D volume image scans. The stimulus runs consisted of four fMRI collection periods for both cutaneous and muscle stimuli, at either the 2T or 4T stimulus intensity followed by a rest period, and with each stimulus intensity presented twice. For each specific stimulus, a boxcar paradigm was used, consisting of 60 dynamic scans (interscan interval 3 sec), divided into six periods, three stimulus periods and three rest periods, with each period having a duration of 30 sec (TR 3000 per dynamic scan). Two different paradigms, A and B, were used, which corresponded to the fMRI collection run commencing with either (A) a rest period (with stimulus onsets at scans 11, 31 and 51) or (B) a stimulus period (stimulus onsets at scans 01, 21 and 41). One of four different protocols was randomly used: AAAA; BBBB; ABAB; BABA.

### Functional MRI experimental data acquisition

All experimental data were acquired using a 3T MR Achieva scanner (Philips) which was equipped with a transmit‐receive head coil. Image artifacts were avoided by stretching the copper stimulation cables as much as possible and by using (nonmagnetic) carbon electrodes. Subjects were supine and head fixation was achieved by two foam cushions and by straps across the head. Subjects were instructed to keep their arms relaxed by their sides. The scanner room was dimly lit and subjects were instructed to relax and close their eyes during the experiment but were not permitted to sleep. Subjects wore hearing protection and were connected via intercom with the control room. The entire study lasted at most 90 min per subject, including setup time.

A T_2_‐weighted multishot (TSE) scan preceded the functional scans with parameters T_R_ 4101.0 ms, T_E_ 120.0 ms, flip angle 90.0°, field of view 230.0 mm, matrix dimensions 256 × 256, 30 contiguous transverse slices and a reconstructed voxel size of 0.45 × 0.45 × 4.0 mm. An approximately 12‐cm thick stack of slices was defined, encompassing transverse slices that covered both the cerebrum and cerebellum. The functional scan consisted of a single shot multiple slice T_2_*‐sensitive echo planar imaging (EPI) sequence, with parameters T_R_ 3000 ms, T_E_ 33 ms, flip angle 90.0°, field of view 230.0 mm, matrix dimension 96 × 128, 30 contiguous slices and a reconstructed voxel size of 1.8 × 1.8 × 4.0 mm. The fMRI signal was based on the blood‐oxygen‐level‐dependent (BOLD) effect. The fMRI sequence incorporated dummy pulses prior to image acquisition to ensure adequate steady‐state imaging. A 3D T_1_‐weighted multishot (TFE) echo scan succeeded the functional scans with parameters T_R_ 6.7 ms, T_E_ 3.0 ms, flip angle 8.0°, field of view 230.0 mm, matrix dimensions 256 × 256, 200 contiguous coronal slices, and a reconstructed voxel size of 0.9 × 0.9 × 0.9 mm.

### Data analysis

The acquired image data were converted to Analyze 7.5 format using a conversion program, MRIconvert program (Version 1.02W: J.Smith, Lewis Centre for NeuroImaging, University of Oregon). All image data were analyzed using the Statistical Parametric Mapping (SPM2: Wellcome Department of Imaging Neuroscience, Institute of Neurology, University College London) software package implemented in MATLAB 6.5.1 (The Mathworks Inc., Natick, MA).

Post processing steps included image realignment, normalization, and smoothing. The scans were first corrected for small motion artifacts by trilinear realignment (quality 0.75) to the first volume scan of each time series. Subsequently, the images were normalized to the fourth image by transformation into standard space, using the EPI template image of the Montreal Neurological Institute (MNI). The voxel sizes of the normalized images were 2 × 2 × 4 mm isotropically with trilinear interpolation and template bounding box (−90:90, −126:90, −72:108). To enhance the spatial signal‐to‐noise ratio and to facilitate intersubject averaging, the normalized images were smoothed with a Gaussian filter with a full width at half maximum of 8 mm. Four of the eight runs from one subject were excluded due to data corruption during acquisition.

Data were analyzed for each condition at both the individual and group level as a General Linear Model for fMRI time series. The fMRI design matrix for each run was specified in scans and vector of onsets, using the hemodynamic response function. Runs were not replications due to the different onsets for each subject. Smoothed data from each run were then scaled before the SPM was estimated. Initially a fixed effects analysis was used but this analysis showed wide interindividual variation for areas of significant sensorimotor activation. A second‐level random effects analysis was, therefore, performed. This technique allows inferences to be made about the population from which the subjects are drawn, rather than just for the group of subjects studied (Penny and Holmes 2007). Because we were primarily interested in the effects of different types of afferents, the main analysis consisted of considering the effects of combined muscle (both 2T and 4T) and skin (both 2T and 4T) stimulation for all subjects. For the main analysis a *P *<**0.001 (*t* = 3.73–3.79) criterion was used, with a minimum cluster size of 5. The active conditions were compared using a random effects analysis using *t‐*tests with a less stringent criterion (*P *<**0.005) but masked by the previously demonstrated significant changes for the condition of interest. The resulting sets of images represented statistical parametric maps of the *t* statistic SPM{*t*}.

The sterotaxic coordinates of Talairach and Tournoux (1988) were used to define the Brodmann areas of the observed activation foci after transformation from MNI coordinates using the Matlab file (mni2tal.m, M. Brett 1999: Cambridge Imagers Unit, Cambridge, UK). The cortical areas corresponding to the observed activation foci were derived from the Talairach daemon client program (Version 2.0; Research Imaging Centre, UTHSCSA). The exact or nearest gray matter structure was reported as the area corresponding to each coordinate. One point initially allocated to area 4 was reassigned to area 3 and one point the reverse after review using the Talairach and Tournoux (1988) atlas and plotting on a representative high‐resolution MRI image. All images were displayed according to radiological convention, that is the right hemisphere is shown on the left side of the image. All tabulated data are shown in MNI coordinates. Cerebellar locations were based upon the atlas of Schmahmann et al. (1999) and for the cerebellar nuclei the work of Dimitrova et al. (2002, 2006).

## Results

### Muscle stimulation (2T and 4T)

**Figure 2.**
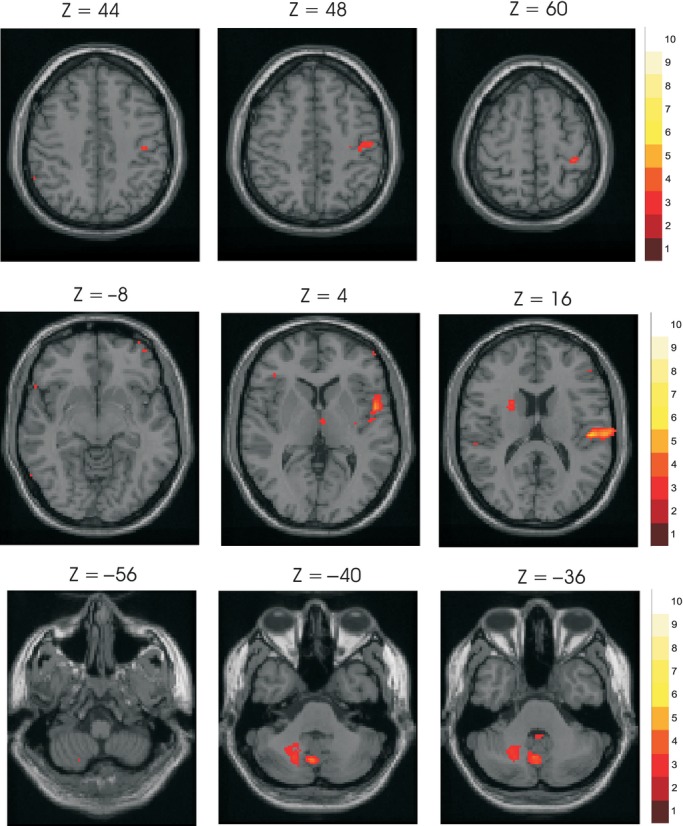
Activations in response to muscle stimulation. Group brain activation in response to stimulation of the motor point of the right first dorsal interosseus muscle at 2 and 4T, using random effects analysis. Statistical images were coregistered with the SPM‐MNI single subject T1 image. The axial/horizontal sections in the upper row show activation areas at the sensorimotor cortex. Sections in the middle row show activation areas in the second sensory area, thalamus, and insula. Sections in the lower row show activation areas in the right and midline cerebellum, including the nucleus (dentate). Colored bar labels indicate levels of activation in *t* value. For illustration purposes a cutoff of *t* = 3.5 has been used.

The random effects analysis produced multiple foci of significant activation ipsilaterally in the cerebellum and predominantly contralaterally for the cerebrum and subcortical areas (Fig. 2, Table 1). Highly significant activations were present in the contralateral insula and nearby temporal lobe. Activation sites were present within the contralateral sensorimotor cortex, in two distinct areas corresponding to heights (*Z*) of 60 and 44–48 mm above the reference plane. Activations were also present within the contralateral superior temporal gyrus, thalamus, and basal ganglia. The significant activations within the cerebellum consisted of a near‐midline group and a second group lying more laterally deep within the ipsilateral cerebellar hemisphere (Table 1).

**Table 1.** Muscle stimulation – random effects. Brain areas activated in response to stimulation of the right first dorsal interosseous muscle nerve (*t*‐test, *P *< 0.001 Uncorrected, Voxels ≥ 5).

Structure/Anatomy	Contralateral (Left)						Ipsilateral (Right)					
	BA	MNI coordinates			SPM {*T*}	No. of voxels	BA	MNI coordinates			SPM {*T*}	No. of voxels
		*x*	*y*	*z*				*x*	*y*	*z*		
Sensorimotor cortex												
Precentral gyrus	**4**	**34**	**−32**	**60**	**4.84**	**18**						
Postcentral gyrus*	3	48	**−**18	48	4.47	27*						
Postcentral gyrus*	3	40	**−**20	44	4.46	27*						
Temporal lobe												
Superior gyrus	42	60	**−**22	12	6.44	209*						
Superior gyrus	22	58	−10	8	4.55	209*						
Superior gyrus	22	50	2	4	5.92	107*						
Inferior gryrus	37	56	−46	−24	4.57	8						
Frontal lobe												
Middle gyrus	10	50	54	0	4.94	8						
Insula												
							13	−28	−6	24	3.99	71*
	13	48	−22	16	6.93	209*						
	**13**	**44**	**−4**	**−4**	**3.96**	107*						
Subcortical												
Claustrum								−24	8	20	4.9	71*
Thalamus		2	−10	4	4.12	6						
Globus pallidus		24	−6	−8	6.82	56*						
Claustrum		34	−12	0	4.04	56*						
Cerebellum												
								**−2**	**−46**	**−36**	**3.95**	**7**
								−6	−66	−40	5.5	81*
								−8	−60	−32	4.29	81*
								−22	−64	−40	4.66	116*
								−26	−58	−36	4.44	116*
								−26	−54	−44	4.22	116*

Combined data for both muscle nerve stimulation intensities. Threshold for statistical significance was *P *<**0.001 (uncorrected) with a cluster size of 5 or more voxels, using second‐level random effects analysis. The number of voxels indicates the overall spatial extent of significant clusters within the activated volume. Cluster sizes with asterisk are foci within that cluster. SPM {*T*} denotes the *t* values for the stereotaxic coordinates. Areas shown in bold had significantly more activated by muscle afferent stimulation than by cutaneous stimulation. Stereotaxic coordinates of peak activation are expressed in millimeters and refer to medial‐lateral position (*x*) relative to midline. (Montreal Neurological Institute coordinates: positive = left), anterior‐posterior position (*y*), relative to the anterior commissure (positive = anterior), and superior‐inferior position (*z*) relative to the commissural line (positive = superior). Localization was based upon transformed Talairach coordinates (see text). BA, Brodmann's area.

* These points were initially allocated to area 4 by the daemon, but review suggested they were postcentral.”

The muscle afferent stimulation condition also evoked significant areas of reduced flow in a large number of locations bilaterally, mainly lying within the posterior parietal and occipital lobes and anterior temporal lobes (Table 2).

**Table 2.** Muscle stimulation – random effects. Brain areas with reduced flow in response to stimulation of the right first dorsal interosseous muscle nerve (*t*‐test, *P *<**0.001 Uncorrected, Voxels ≥5).

Structure/Anatomy	Contralateral (Left)						Ipsilateral (Right)					
	BA	MNI coordinates			SPM {*T*}	No. of voxels	BA	MNI coordinates			SPM {*T*}	No. of voxels
		*x*	*y*	*z*				*x*	*y*	*z*		
Sensorimotor cortex/Frontal lobe												
Precentral gyrus							4	−22	−28	76	−4.41	18*
Precentral gyrus							4	−16	22	76	−4.37	18*
Inferior frontal gyrus							47	−28	18	−28	−5.36	89*
Parietal lobe												
Superior parietal lobule							**7**	**−4**	**−70**	**60**	**−5.2**	197*
Precuneus							7	−2	−52	68	−5.19	197*
Precuneus							7	−2	−60	52	−4.89	197*
Superior parietal lobule	7	8	−68	60	−4.66	10						
Precuneus	**7**	**18**	**−80**	**48**	**−4.14**	**8**						
Limbic lobe												
Posterior cingulate							31	−2	−68	12	−4.21	5
Posterior cingulate	29	2	−46	4	−4.04	10						
Parahippocampal gyrus							30	−12	−42	−12	−5.9	80*
Parahippocampal gyrus							**28**	**−20**	**−16**	**−28**	**−4.64**	**21**
Parahippocampal gyrus							36	−28	−34	−28	−4.3	7
Parahippocampal gyrus	**36**	**30**	**−34**	**−28**	**−5.48**	96*						
Temporal lobe												
Superior temporal gyrus							21	−60	−14	−4	−5.6	12
Superior temporal gyrus	22	68	−22	0	−4.42	31*						
Superior temporal gyrus	**38**	**34**	**6**	**−20**	**−4.35**	**5**						
Superior temporal gyrus	**38**	**44**	**22**	**−24**	**−5.17**	**10**						
Superior temporal gyrus							**38**	**−28**	**10**	**−32**	**−6.01**	89*
Superior temporal gyrus							38	−30	2	−32	−4.87	89*
Superior temporal gyrus	38	42	16	−40	−4.28	13						
Middle temporal gyrus	**21**	**68**	**−12**	**−4**	**−5.19**	31*						
Middle temporal gyrus	**21**	**58**	**0**	**−12**	**−5.33**	56*						
Middle temporal gyrus	**21**	**60**	**8**	**−16**	**−4.29**	56*						
Middle temporal gyrus							21	−42	2	−36	−4.09	14*
Middle temporal gyrus							21	−50	6	−40	−4.02	14*
Fusiform gyrus	37	34	−64	−24	−3.99	6						
Occipital lobe												
Lingual gyrus	**19**	**12**	**−48**	**−4**	**−4.26**	**6**						
Fusiform gyrus							**37**	**−22**	**−50**	**−16**	**−4.31**	80*
Fusiform gyrus							**19**	**−24**	**−64**	**−16**	**−3.94**	**5**
Fusiform gyrus	**19**	**20**	**−54**	**−20**	**−5.32**	95*						
Cerebellum												
		24	−54	−28	−4.57	95*						
		20	−64	−24	−4.06	95*						
		**36**	**−44**	**−32**	**−5.92**	96*						
								−34	−40	−36	−5.09	16

Combined data for both muscle nerve stimulation intensities – reductions. Threshold for statistical significance was *P *<**0.001 (uncorrected) with a cluster size of 5 or more voxels, using second level random effects analysis. Number of voxels indicates the overall spatial extent of significant clusters within the activated volume. Cluster sizes with asterisk are foci within that cluster. SPM {*T*} denotes the *t* values for the stereotaxic coordinates. Areas shown in bold had significantly greater flow reductions with muscle afferent stimulation than with cutaneous stimulation. Stereotaxic coordinates of peak activation are expressed in millimeters and given in MNI space. Localization was based upon transformed Talairach coordinates (see text). BA, Brodmann's area.

### Cutaneous stimulation (2T and 4T)

**Figure 3.**
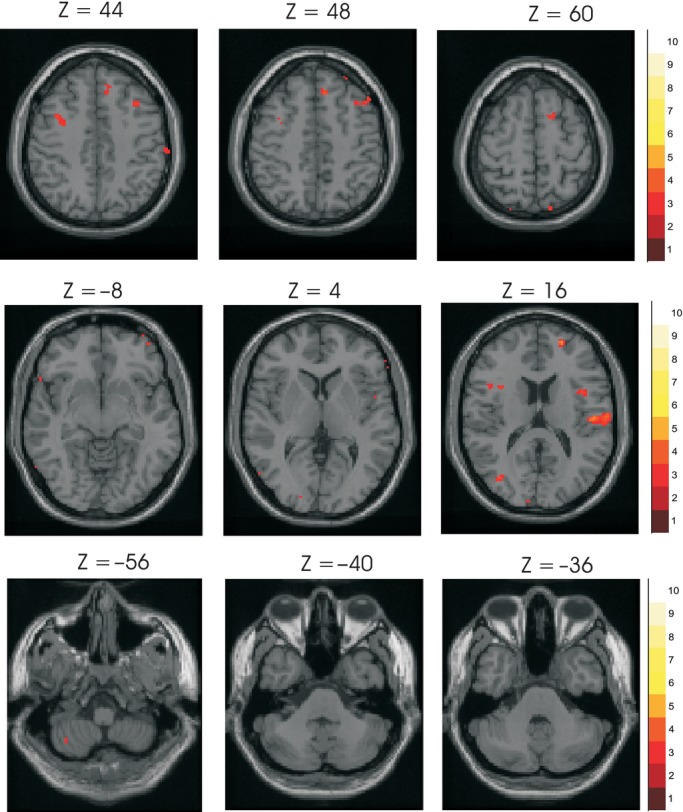
Activations in response to cutaneous stimulation. Group brain activation in response to stimulation of cutaneous afferents of the right index finger at 2 and 4T, using random effects analysis. Statistical images were coregistered with the SPM‐MNI single subject T1 image. The axial/horizontal sections in the upper row show frontal and parietal activation areas. Sections in the middle row show areas of activation including the second sensory area (SII). Sections in the lower row show an area of activation within both the right cerebellum. Colored bars labels indicate levels of activation in *t* value. For illustration purposes a cutoff of *t* = 3.5 has been used.

Cutaneous stimulation activated a wide range of areas, predominantly in the contralateral hemisphere (Fig. 3, Table 3). Multiple areas of activation within the frontal lobes were present, including one area of activation present in the contralateral sensorimotor cortex at height 44 mm. Although initially allocated by the Talairach daemon client program to area 4, review suggested this point was post central, on the crown of the gyrus within area 3. Contralateral parietal lobe activation corresponding to the second somatosensory cortex (SII: area 40) was present and there was activation within the insula (area 13). Bilateral areas of activation were present in the temporal lobes. The cerebellum showed an area of activation ipsilaterally with other weaker areas of activation, below our statistical criterion.

**Table 3.** All cutaneous stimulation excitation (random effects). Brain areas activated in response to suprathreshold stimulation of the right index cutaneous afferents (*t*‐test, *P *<**0.001 Uncorrected).

Structure/Anatomy	Contralateral (Left)						Ipsilateral (Right)					
	BA	MNI coordinates			SPM {*T*}	No. of voxels	BA	MNI coordinates			SPM {*T*}	No. of voxels
		*x*	*y*	*z*				*x*	*y*	*z*		
Frontal lobes												
Superior frontal gyrus	6	10	12	56	4.5	17						
Middle frontal gyrus	8	40	20	52	6.23	38*						
Precentral gyrus	9	34	20	44	4.6	38*						
Superior frontal gyrus	8	6	30	48	4.96	26*						
Medial frontal gyrus	8	6	38	44	4.11	26*						
Precentral gyrus							**9**	**−38**	**8**	**44**	**4.5**	**18**
Postcentral gyrus*	3	62	−22	44	4.07	7						
Inferior frontal gyrus							44	−46	10	20	4.14	17
Superior frontal gyrus	10	20	54	16	5.68	26*						
Superior frontal gyrus	10	12	62	20	4.24	26*						
Inferior frontal gyrus	46	58	38	8	4.66	13*						
Inferior frontal gyrus	47	58	36	0	3.9	13*						
Parietal lobes												
Superior parietal lobule	**7**	**12**	**−78**	**60**	**4.58**	**6**						
Supramarginal gyrus	40	46	−52	32	5.05	6						
Temporal lobes												
Middle temporal gyrus	39	36	−70	28	4.7	18*						
Superior temporal gyrus	39	40	−62	24	3.97	18*						
Transverse temporal gyrus	42	62	−16	16	5.19	67*						
Middle temporal gyrus							**39**	**−38**	**−74**	**16**	**4.83**	**56** *
Middle temporal gyrus							**39**	**−58**	**−68**	**8**	**4.58**	**56** *
Middle temporal gyrus							**37**	**−60**	**−66**	**0**	**4.5**	**56** *
Insula												
Insula	13	48	−18	16	5.47	67*						
Insula	13	40	6	16	4.6	11						
Limbic lobe												
Parahippocampal gyrus	28	16	−4	−12	4.83	10						
Cerebellum												
								−36	−60	−56	4.1	5

Random effects model, second‐level analysis. *P *<**0.001, minimum cluster size = 5. Areas shown in bold were significantly more activated by cutaneous stimuli than with muscle nerve stimulation. Stereotactic coordinates of peak activations are in millimeters and given in MNI space. BA, Brodmann's area.

* This point was initially allocated to area 4 by the daemon.

Areas of significantly reduced flow were also present (Table 4), but substantially fewer than for muscle stimulation.

**Table 4.** All cutaneous stimulation. Brain areas with reduced flow in response to stimulation of the right index cutaneous afferents (*t*‐test, *P *<**0.001 Uncorrected, Voxels ≥5).

Structure/Anatomy	Contralateral (Left)						Ipsilateral (Right)					
	BA	MNI coordiates				No. of voxels	BA	MNI coordinates			SPM {*T*}	No. of voxels
		*x*	*y*	*z*	SPM {*T*}			*x*	*y*	*z*		
Temporal lobe												
Superior temporal gyrus	38	46	10	−20	−5.56	27						
Limbic Lobe												
Parahippocampal gyrus	**35**	**−12**	**−32**	**−16**	**−4.95**	83*						
Parahippocampal gyrus							30	6	−38	−8	−4.12	83*
Subcortical												
Thalamus		−8	−32	−8	−4.12	83*						

Combined data for both cutaneous nerve stimulation intensities showing reductions. Threshold for statistical significance was *P *<**0.001 (uncorrected) with a cluster size of 5 or more voxels, using second level random effects analysis. Number of voxels indicates the overall spatial extent of significant clusters within the activated volume. Cluster sizes with asterisk (*) are foci within a larger cluster. SPM {*T*} denotes the *t* values for the stereotaxic coordinates. The area shown in bold had a significantly greater flow reduction with cutaneous stimulation than with muscle nerve stimulation. Stereotaxic coordinates of peak activation are expressed in millimeters and given in MNI space. Localization was based upon transformed Talairach coordinates (see text). BA, Brodmann's area.

### Inter‐stimulus comparisons

#### Muscle versus cutaneous

The higher site of sensorimotor cortex activation, activation in the contralateral insula, and activation in the ipsilateral cerebellum were significantly greater for muscle nerve stimulation than for cutaneous nerve stimulation (shown in bold in Table 1). Cutaneous stimulation was associated with several areas of significantly increased activation compared to muscle afferent stimulation, including one site within the contralateral superior parietal lobule. The other sites lay within the ipsilateral hemisphere and included the ipsilateral precentral gyrus and multiple sites within the middle temporal gyrus (Table 3).

Muscle afferent activation evoked significantly greater inhibition than cutaneous stimulation at multiple sites within the parietal lobes, parahippocampal gyri, temporal, occipital lobes, and one site in the cerebellum (Table 2). Cutaneous stimulation caused significantly more inhibition at a single site in the contralateral parahippocampal gyrus (Table 4).

### 

#### 4T versus 2T: muscle and cutaneous

Muscle 4T stimulation evoked significantly greater activation (*t* = 3.51–8.0) than 2T stimulation after masking, for five of the significant six sites in the ipsilateral cerebellum, the sites in the contralateral thalamus, globus pallidus, claustrum, insula, and one more superior site, in the postcentral cortex (Table 1). Sites within the ipsilateral parietal lobe, bilateral temporal lobes, contralateral fusiform gyrus, and ipsilateral cerebellum were significantly more inhibited with 4T compared to 2T stimulation.

No points were significantly more activated or inhibited by 4T cutaneous stimulation than with 2T within the significant regions of cutaneous activation.

## Discussion

In this study, motor threshold was used as a substitute for muscle afferent threshold in contrast to the perceptual threshold used for cutaneous stimulation. Assuming that electrical threshold is inversely related to fiber diameter (Erlanger and Gasser 1937), it is likely that the 2T electrical stimulus activated most of the Group I muscle afferent fibers and A*β* fibers. 4T stimulation would have activated most of the Group II and some Group III fibers, as well as the A*δ* fibers. The stimulus also activated motor axons and the ensuing muscle contraction would both directly and indirectly evoke muscle afferent volleys, supplementing the direct effects. Muscle afferents of Group I and II transduce muscle length and force and have important roles in proprioception (e.g., Proske 2006; Proske and Gandevia 2012) and the regulation of muscle activation both segmentally and supraspinally. Activation of cutaneous afferents, at 2 and 4 times perceptual threshold, was achieved by using digital nerve stimulation although some joint receptors were probably included. The largest cutaneous afferents mediate sensations of touch and vibration (Mountcastle 1974a). Projections of the large diameter primary afferents ascend to the thalamus via the dorsal column‐medial lemniscus and other pathways, the cerebellum via (for the upper limbs) the cuneocerebellar and rostral spinocerebellar and other tracts and brainstem targets (McIntyre 1974; Sengul and Watson 2012).

Selective electrical stimulation of both cutaneous and muscle afferent groups produced statistically significant increases in regional blood flow within cortical, subcortical, and cerebellar regions. Given we used single stimuli, likely to excite large diameter afferents, the areas of activation would be expected to coincide with those activated by cutaneous or muscle afferents with strong connectivity via oligosynaptic pathways. Responses corresponding to these projections might also be expected to be elicited by cutaneous stimulation or imposed movement under experimental conditions in intact animals or humans. The patterns of activation evoked by stimulating the two types of afferents differed. The cortical areas activated with muscle stimulation included many associated with the generation of movement, including primary motor cortex (MI), thalamus, basal ganglia and cerebellum, as well as the insula, and other areas of the frontal and temporal lobes. Cutaneous stimulation activated multiple areas in frontal, parietal, and temporal lobes, insula, parahippocampal gyrus, and cerebellum.

### Sensorimotor cortex

Three significant foci of activation were present in the contralateral sensorimotor cortex following muscle afferent stimulation, with at least two distinct areas. MI lies in the precentral gyrus (area 4) and receives inputs from multiple areas including the premotor and supplementary motor area (SMA) (area 6). The activation evoked by passive proprioceptive stimulation has been found to involve fewer cortical motor areas than active movement (Weiller et al. 1996; Mima et al. 1999) and in this study we, like these authors, found no areas of activation within nonprimary motor cortex such as SMA and premotor cortex. Brinkman and Porter (1979) reported that responses to passive movement were infrequent in SMA, at longer latency and insecure compared to those to the motor cortex. Naito et al. (2005) did find activation of nonprimary motor areas using muscle vibration, a process they suggested was due to muscle spindle afferent information being transmitted from “primary” motor and sensory areas to “non‐primary” ones. This may have been facilitated by the longer periods of continuous stimulation used (32s), allowing temporal summation of effects.

Cortical activation following mixed nerve stimulation has been reported previously. Ibáñez et al. (1995) showed single focus in S1 following median nerve stimulation at a similar height to our lower pair of foci. Although the projection to area 3a is the densest cortical representation of muscle afferents, short latency projections to MI have been also been described (Colebatch et al. 1990). The MI representation for first dorsal interosseous muscle lies within the hand area of the precentral gyrus and this can be localized by its characteristic appearance of a “knob” (Yousry et al. 1997). Spiegel et al. (1999) used a sensory evoked potential paradigm to stimulate the median nerve at motor threshold and produced two distinct contralateral foci, lying in MI and SI. The center of activated cortex in MI lay 5 mm superior to the activated cortex in SI. Our most superior focus lay within the hand “knob” area for the right hemisphere of the image ch2 (Montreal Neurological Institute) and thus it is likely to represent activation within M1 by muscle afferents whereas the lower foci are likely to lie within S1. Our separation is greater than that reported by Spiegel et al. (1999) but we have confirmed that at least two separate sites of muscle afferent projections are present within the sensorimotor cortex, the superior one of which corresponds to the hand area of M1 as defined anatomically.

We found that the M1 activation is significantly more powerful for muscle afferents than for cutaneous afferents. One area of significant activation associated with cutaneous stimulation lay within SI at the crown of the postcentral gyrus and thus in area 3b. Electrophysiological data suggest that the subdivisions of SI have different functional roles. Neurons in areas 3a and 2 respond to stimulation of deep receptors (Group I & II muscle afferents), whereas neurons in areas 3b and 1 exhibit a predominant responsiveness to stimulation of cutaneous receptors, consistent with our findings (Mountcastle 1974b; Allison et al. 1991; Iwamura et al. 1993).

### Areas activated with muscle stimulation only

Muscle afferent stimulation was associated with activations in the thalamus and basal ganglia. The two areas mapped as the claustrum may represent extensions of the bilateral insular activations and will not be considered further. Muscle afferents from the upper limbs ascend in the dorsal columns, synapse in the cuneate nucleus and then terminate in the ventroposterior lateral (VPL) nucleus of the thalamus (Brodal 1981), consistent with the activation we found. The major projection from this nucleus is to postcentral sensory cortex – areas 1, 2, 3 and 5 (Macchi and Jones 1997; Mai and Forutan 2012). Parts of the “motor” thalamus, which projects to precentral motor areas, contain cells which respond to passive movements and muscle stretch at short latency (Vitek et al. 1994). Recordings from Vim in awake humans also demonstrate responses to active and passive joint movements, with neurons having characteristics consistent with muscle afferent input (Ohye et al. 1989).

The contralateral globus pallidus showed activation with muscle afferent stimulation. The pallidum projects to the VLa (VLo) area of the thalamus (Macchi and Jones 1997) and labeling studies have shown projections from these specific thalamic nuclei to the striatum (McFarland and Haber 2001). In the somatosensory part of the GPi, up to 25% of neurones respond to kinesthetic stimuli in recordings made from patients operated on for Parkinson's disease (Lozano and Hutchison 2002). In monkeys, 22 and 37% of movement‐related neurones in GPe and GPi respond to passive joint movement, but very few respond to cutaneous stimuli (DeLong et al. 1985).

### Areas activated by cutaneous stimulation only

Cutaneous stimuli, unlike muscle afferent activation, were associated with activation within the contralateral parahippocampal gyrus, bilateral temporal foci, contralateral parietal lobe, and multiple frontal areas, including ipsilateral sites. A number of studies have shown that cutaneous electrical stimulation can activate cortical areas in addition to the primary sensory cortex, including the contralateral superior parietal lobule (area 7), SMA/CMA, and insula as well as bilateral inferior parietal lobule (areas 39 and 40: Forss et al. 1994a; Ruben et al. 2001; Deuchert et al. 2002). The superior and inferior parietal lobules are considered to be multimodal association cortex and show widespread connectivity (Caspars et al. 2012).

### Cerebellum

The cerebellum was strongly activated ipsilaterally in this study following muscle afferent stimulation. High threshold muscle afferent activation was significantly more effective than low‐intensity stimulation for nearly all the ipsilateral foci, supporting a role for higher threshold afferents. The cerebellum has a role in motor learning (Jenkins et al. 1994) and the ongoing modulation of motor output including timing of muscle contractions (Holmes 1952). Cerebellar activation has been shown previously using electrical stimulation of cutaneous and mixed nerves (e.g., Eccles et al. 1968). Painful intramuscular electrical stimulation of the brachioradialis muscle can produce ipsilateral activation in the declive of cerebellum, whereas painful cutaneous laser stimulation activates ipsilateral culmen (Svensson et al. 1997). Backes et al. (2000) showed significant activation in SI and the ipsilateral cerebellum with maximum current stimulation of the median nerve. Takanashi et al. (2003) reported cerebellar activation following stimulation of cutaneous afferents in the finger or toe. Muscle and other afferents project to the cerebellum through the dorsal and ventral spinocerebellar tracts and their forelimb equivalents mainly to the intermediate and midline divisions of the cortex (Brodal 1981). Group 1a, 1b, and Group II muscle afferent fibers project to neurons within both the dorsal spinocerebellar tract (DSCT) and ventral spinocerebellar tracts (VSCT) while the latter also receives input from high threshold (II and III) afferents of muscle, joint and cutaneous origin. While the DSCT terminates ipsilaterally, afferents within the VSCT ascend in the contralateral cord and terminate bilaterally (McIntyre 1974). Like Grodd et al. (2001) who studied voluntary movement, we found unilateral activation with both muscle and cutaneous stimuli and, like their report, there were at least two distinct foci of activation within the ipsilateral cerebellum – one within the vermis and one more lateral. The latter was more difficult to localize and lay at the lateral boundary of the dentate nucleus, at the base of lobule VI (Dimitrova et al. 2002, 2006; Schmahmann et al. 1999).

Short latency inputs from muscle and cutaneous afferents excite cerebellar neurons (Thach 1967; Eccles et al. 1968, 1971). Muscle afferents caused significantly greater activation than cutaneous afferents in this study. This is consistent with the cerebellum's role in movement but contrasts with some previous reports using direct neuronal recordings from a similar region to our lateral activation zone (paravermal lobules V and VI), where responses to probable cutaneous afferents (tapping) are more easily demonstrated using natural stimulation (Harvey et al. 1977). These observations do not exclude muscle afferents arising from Golgi tendon organs (Iosif et al. 1972; Ishikawa et al. 1972) or high threshold muscle afferents, both of which are unlikely to be activated by passive movements. Our findings indicate that peripheral afferent discharges are likely to contribute significantly to cerebellar activation occurring with voluntary movement.

### Insula

The insula and surrounding somatosensory areas have been previously included as part of SII, the secondary somatosensory area, but this has now been subdivided (Kaas 2012). Activation within the insula region – the cortex lying in the floor of the lateral sulcus, occurred with both cutaneous and muscle afferent stimulation. It was contralateral only for cutaneous stimulation but bilateral for muscle afferent stimulation. It was significantly greater following muscle than cutaneous afferent stimulation, and significantly greater for 4T than 2T stimulation implying a role for Group II and probably Group III afferents. The posterior insula is a multisensory area (Mazzola et al. 2006) and stimulation can evoke painful sensations (Ostrowsky et al. 2002; Afif et al. 2008). It may code the magnitude of sensory inputs (Baliki et al. 2009). We did not match our stimuli for their subjective discomfort and the 4T muscle stimulation was considered to be the most painful.

Previous imaging studies have looked at the involvement of muscle and cutaneous afferents in pain. Svensson et al. (1997) used high‐frequency intramuscular electrical stimulation (brachioradialis m.) and cutaneous stimulation with PET imaging to observe regional activation in a variety of mainly contralateral cortical regions which overlapped for the two modalities. They reported that rCBF in the anterior insular cortex increased with noxious cutaneous stimulation. Electrical stimulation of cutaneous digital afferents may activate contralateral SI and SII areas with somatotopic representation (Deuchert et al. 2002). A study by Niddam et al. (2002) compared painful to non‐painful intramuscular electrical stimulation using event‐related fMRI (3T), and found that painful muscle stimuli increased activation in ipsilateral SII and IPL (areas 43 & 40), middle and posterior insula as well as contralateral middle frontal gyrus (area 10), precentral gyrus (area 44), superior temporal gyrus (area 22), limbic lobe (areas 23, 24 and 32), anterior and posterior insula, and caudate body and tail. Activation within the posterior insular cortex was significantly greater for painful muscle stimulation.

### Temporal areas

Areas within the middle and upper temporal lobes were activated during both muscle and cutaneous afferent stimulation. Area 42 and area 41 correspond to Heschl's gyrus, the primary auditory field (Brodal 1981). Areas 22, 37 and 39, all showing activation, are perisylvian language areas. None of these areas is directly related to somatosensory input, but they have shown activation in previous studies. Niddam et al. (2002) reported bilateral activation within area 22 (right predominant) in response to both non painful and painful muscle stimulation, an effect that attributed to stimulus “salience”. In this study, for muscle afferent stimulation, there was a clear left‐sided predominance. As fMRI imaging is noisy and semirhythmical, it is possible that the increased activation in these areas represented heightened awareness of external stimuli during the period of stimulation.

### Relationship with evoked potentials

Ironically, given the wide range of sites of activation, early studies using evoked potentials showed responses in cerebral cortex only when stimuli engaged Group II or Group III volleys, leading to the conclusion that Group I activity alone was ineffective (McIntyre 1974). In contrast, afferent volleys from skin and joint nerves readily elicited cortical potentials. Gandevia and Burke (1988, 1990), using motor point and intrafascicular stimulation, reported evoked potentials for both proximal and distal muscles of the arm, but the potentials were small compared to those for a digital nerve. Allison et al. (1991) concluded that short latency SEPs evoked by stimulation of the median, a mixed nerve, were primarily the result of cutaneous fibers and that muscle afferents contributed little. Our findings suggest that evoked potentials are not a reliable means by which to assess the overall cortical projections of afferent species. The larger evoked responses to cutaneous afferents are likely to be due in part to the location and orientation of the generators, the relative number of cutaneous afferents and perhaps the synchrony of the volley. Evoked potentials will be biased toward cortical responses whereas here we have compared muscle and cutaneous afferent effects at the cortical, subcortical, and cerebellar levels.

The relationship between changes in BOLD signal, as recorded here, and electrophysiological measures has been the subject of a number of investigations. EEG potentials are believed to arise from summed postsynaptic potentials and horizontally oriented cortical cells, and subcortical structures contribute little, if anything, to the scalp‐recorded EEG (Aminoff 1999). Likewise, simultaneous recordings in the visual cortex of anesthetized monkeys have shown that BOLD signal increases reflect increases in neural activity, which is more closely related to local field potentials (LFPs), a measure of afferent input, than to neuronal discharges (Logothetis et al. 2001). This may apply particularly to high‐frequency LFPs (100–150 Hz, Ojemann et al. 2010) but may vary for different cortical regions (Huettel et al. 2004). Our findings indicate a widespread influence of muscle afferents, including in the sensory cortex, which is more intense than that for cutaneous afferents. The effects are not simply increases in areas of BOLD activation, as muscle afferents also cause significantly larger areas of reduced activation than cutaneous afferents. Cortico‐cortical inhibition of nonsalient sensory receptive areas is a general feature of sensory processing and appears to be mediated by GABAergic transmission (Iurilli et al. 2012).

### Functional significance

Studies using active movement must go to considerable lengths to avoid differences in the kinematics of the movements so as not to confound their results by differences in the peripheral afferent activations evoked by the movements themselves (e.g., Schaechter and Perdue 2008). Disorders of sensorimotor integration are common in movement disorders (Tempel and Perlmutter 1990; Abbruzzese and Berardelli 2003). In dystonic disorders, sensory input may either trigger or improve the abnormality and the widespread projections we have demonstrated, including the basal ganglia, could underlie these phenomena. Clinically, it is widely accepted that normal movement is not possible in the absence of proprioceptive sensation and deafferented patients typically perform ataxic movements (“sensory ataxia” – e.g., Holmes 1952) and this may be explained by the widespread projections of muscle afferents to motor‐related areas interacting with the motor command. Our findings also support Holmes' observation that it is not simply a loss of conscious proprioception that underlies the clinical deficits but also the concomitant loss of the subconscious afferent input to the cerebellum and other areas. Functional imaging indicates that the central effects of muscle afferents are more widespread and more focused on the motor system than are those of cutaneous afferents. The same applies to projections to sensorimotor cortex but is contrary to the evidence that might be deduced from evoked potentials.

## Acknowledgments

The authors thank Professor John Watson, Dr Luke Henderson, Dr Nathan Walters, Ms Kathy Hughes, and Dr Michael Hornberger for technical assistance.

## Conflict of Interest

None declared.

## References

[b1] Abbruzzese G. Berardelli A. 2003 Sensorimotor integration in movement disorders. Mov. Disord.; 18:231-240 12621626 10.1002/mds.10327

[b2] Afif A. Hoffman D. Minotti L. Benabid A. L. Kahane P. 2008 Middle short gyrus of the insula implicated in pain processing. Pain; 138:546-555 18367333 10.1016/j.pain.2008.02.004

[b3] Allison T. McCarthy G. Wood C. C. Jones S. J. 1991 Potentials evoked in human and monkey cerebral cortex by stimulation of the median nerve. A review of scalp and intracranial recordings. Brain; 114:2465-2503 1782527 10.1093/brain/114.6.2465

[b4] Aminoff M. J. 1999In: Aminoff M. J. (ed.). Electroencephalography: general principles and clinical applications. Electrodiagnosis in clinical neurology4th edn. New York Churchill Livingstone

[b5] Backes W. H. Mess W. H. Van Kranen‐Mastenbroek V. Reulen J. P. 2000 Somatosensory cortex responses to median nerve stimulation: fMRI effects of current amplitude and selective attention. Clin. Neurophysiol.; 111:1738-1744 11018487 10.1016/s1388-2457(00)00420-x

[b6] Baliki M. N. Geha P. Y. Apkarian A. V. 2009 Parsing pain perception between nocicieptive representation and magnitude estimation. J. Neurophysiol.; 101:875-887 19073802 10.1152/jn.91100.2008 PMC3815214

[b7] Brinkman C. Porter R. 1979 Supplementary motor are in the monkey: activity of neurons during performance of a learned motor task. J. Neurophysiol.; 423:681-709 107282 10.1152/jn.1979.42.3.681

[b8] Brodal A. 1981 Neurological anatomy3rd edn. New York Oxford Univ. Press

[b9] Caspars S. Amunts K. Zilles K. 2012 1036-1055In: Mai J. K. Paxinos G. (eds.). Posterior parietal cortex: multimodal association cortex. The human nervous system Amsterdam Academic Press

[b10] Colebatch J. G. Sayer R. J. Porter R. White O. B. 1990 Responses of monkey precentral neurones to passive movements and phasic muscle stretch: relevance to man. Electroenceph. Clin. Neurophysiol.; 75:44-55 1688773 10.1016/0013-4694(90)90151-9

[b11] Collins D. F. Refshauge K. M. Gandevia S. C. 2000 Sensory integration in the perception of movements at the human metacarpophalngeal joint. J. Physiol. (Lond.); 529:505-515 11101658 10.1111/j.1469-7793.2000.00505.x PMC2270207

[b12] DeLong M. R. Crutcher M. D. Georgopoulos A. P. 1985 Primate globus pallidus and subthalamic nucleus: functional organization. J. Neurophysiol.; 53:530-543 3981228 10.1152/jn.1985.53.2.530

[b13] Deuchert M. Reuben J. Schwiemann J. Meyer R. Thees S. Krause T. 2002 Event‐related fMRI of the somatosensory system using electrical finger stimulation. NeuroReport; 13:365-369 11930139 10.1097/00001756-200203040-00023

[b14] Dimitrova A. Weber J. Redies C. Kindsvater K. Mashke M. Kolb F. P. 2002 MRI atlas of the human cerebellar nuclei. Neuroimage; 17:240-255 12482081 10.1006/nimg.2002.1124

[b15] Dimitrova A. Zeljko D. Schwarze F. Maschke M. Gerwig M. Frings M. 2006 Probabilistic 3D MRI atlas of the human cerebellar dentate/interposed nuclei. Neuroimage; 30:12-25 16257240 10.1016/j.neuroimage.2005.09.020

[b16] Eccles J. C. Provini L. Strata P. Táboříková H. 1968 Analysis of electrical potentials evoked in the cerebellar anterior lobe by stimulation of hindlimb and forelimb nerves. Exp. Brain Res.; 6:171-194 5712699 10.1007/BF00235123

[b17] Eccles J. C. Faber D. S. Murphy J. T. Sabah N. H. Táboříková H. 1971 Afferent volleys in limb nerves influencing impulse discharges in cerebellar cortex. II. In Purkynĕ cells. Exp. Brain Res.; 13:36-53 5570422

[b18] Erlanger J. Gasser H. S. 1937

[b19] Forss N. Hari R. Salmelin R. Ahonen A. Hamalainen M. Kajola M. 1994a Activation of the human posterior parietal cortex by median nerve stimulation. Exp. Brain Res.; 99:309-315 7925811 10.1007/BF00239597

[b20] Forss N. Salmelin R. Hari R. 1994b Comparison of somatosensory evoked fields to airpuff and electric stimuli. Electroenceph. Clin. Neurophysiol.; 92:510-517 7527769 10.1016/0168-5597(94)90135-x

[b21] Gandevia S. C. Burke D. 1988 Projection to the cerebral cortex from proximal and distal muscles in the human upper limb. Brain; 111:389-403 2837308 10.1093/brain/111.2.389

[b22] Gandevia S. C. Burke D. 1990 Projection of thenar muscle afferents to the frontal and parietal cortex of human subjects. Electroenceph. Clin. Neurophysiol.; 77:353-361 1697527 10.1016/0168-5597(90)90057-k

[b23] Grodd W. Hűlsmann E. Wildgruber D. Erb M. 2001 Sensorimotor mapping of the human cerebellum: fMRI evidence of somatotopic organization. Hum. Brain Mapp.; 13:55-73 11346886 10.1002/hbm.1025 PMC6871814

[b24] Harvey R. J. Porter R. Rawson J. A. 1977 The natural discharges of Purkinje cells in the paravermal regions of lobules V and VI of the monkey's cerebellum. J. Physiol.; 271:515-536 411917 10.1113/jphysiol.1977.sp012012 PMC1353584

[b25] Holmes G. 1952 Introduction to clinical neurology2nd edn. London Livingstone

[b26] Horne M. K. Porter R. 1980 The discharges during movement of cells in the ventrolateral thalamus of the conscious monkey. J. Physiol.; 304349–372 10.1113/jphysiol.1980.sp013328 PMC1282934 7441539

[b27] Huettel S. A. McKeown M. J. Song A. W. Hart S. Spencer D. D. Allison T. 2004 Linking hemodynamic and electrophysiological measures of brain activity: evidence from functional MRI and intracranial field potentials. Cereb. Cortex; 14:165-173 14704213 10.1093/cercor/bhg115

[b28] Ibáñez V. Deiber M. P. Sadato N. Toro C. Grissom J. Woods R. P. 1995 Effects of stimulus rate on regional cerebral blood flow after median nerve stimulation. Brain; 118:1339-1351 7496791 10.1093/brain/118.5.1339

[b29] Iosif G. Pompeiano O. Strata P. Thoiden U. 1972 The effect of stimulation of spindle receptors and Golgi tendon organs on the cerebellar anterior lobe. II Responses of Purkinje cells to sinusoidal stretch or contraction of hindlimb extensor muscles. Arch. Ital. Biol.; 110:502-542

[b30] Ishikawa K. Kawaguchi S. Rowe M. J. 1972 Actions on afferent impulses from muscle receptors on cerebellar Purkynĕ cells. II. Responses to muscle contraction: effects mediated via the climbing fiber pathway. Exp. Brain Res.; 16:104-114 4265012 10.1007/BF00233377

[b31] Iurilli G. Ghezzi D. Olcese U. Lassi G. Nazzaro C. Tonini R. 2012 Sound‐driven synaptic inhibition in primary visual cortex. Neuron; 73:814-828 22365553 10.1016/j.neuron.2011.12.026 PMC3315003

[b32] Iwamura Y. Tanaka M. Sakamoto M. Hikosaka O. 1993 Rostrocaudal gradients in the neuronal receptive field complexity in the finger region of the alert monkey's post central gyrus. Exp. Brain Res.; 92:360-368 8454001 10.1007/BF00229023

[b33] Jenkins I. H. Brooks D. J. Nixon P. D. Frackowiak R. S. J. Passingham R. E. 1994 Motor sequence learning: a study with positron emission tomography. J. Neurosci.; 14:3775-3790 8207487 10.1523/JNEUROSCI.14-06-03775.1994 PMC6576955

[b34] Kaas J. H. 2012 1074-1109 *in*In: Mai J. K. Paxinos G. (eds.). Somatosensory system. The human nervous system3rd edn. London Academic Press

[b35] Krause T. Kurth R. Ruben J. Schwiemann J. Villringer K. Deuchert M. 2001 Representational overlap of adjacent fingers in multiple areas of human primary somatosensory cortex depends on electrical stimulus intensity: an fMRI study. Brain Res.; 899:36-46 11311865 10.1016/s0006-8993(01)02147-3

[b36] Landgren S. Silfvenius H. Wolsk D. 1967 Somato‐sensory paths to the second cortical projection area of the group I muscle afferents. J. Physiol.; 191:543-559 4860991 10.1113/jphysiol.1967.sp008267 PMC1365491

[b37] Lemon R. N. Porter R. 1976 Afferent input to movement‐related precentral neurons in conscious monkeys. Proc. R Soc. Lond. Biol. Sci.; 194:313-339 11491 10.1098/rspb.1976.0082

[b38] Logothetis N. K. Pauls J. Augath M. Trinath T. Oeltermann A. 2001 Neurophysiological investigation of the basis of the fMRI signal. Nature; 412:150-157 11449264 10.1038/35084005

[b39] Lozano A. M. Hutchison W. D. 2002 Microelectrode recordings in the pallidum. Mov. Disord.; 17Suppl 3:S150-S154 11948770 10.1002/mds.10157

[b40] Macchi G. Jones E. G. 1997 Toward an agreement on terminology of nuclear and subnuclear divisions of the motor thalamus. J. Neurosurg.; 86:670-685 9120632 10.3171/jns.1997.86.4.0670

[b41] Mai J. K. Forutan F. 2012 618-677 *in*In: Mai J. K. Paxinos G. (eds.). Thalamus. The human nervous system Amsterdam Academic Press

[b42] Marani E. E. Lakke A. J. F. 2012 82-140 *in*In: Mai J. K. Paxinos G. (eds.). Peripheral nervous system topics. The human nervous system Amsterdam Academic Press

[b43] Mazzola L. Isnard J. Mauguière F. 2006 Somatosensory and pain responses to stimulation of the second somatosensory area (SII) in humans. A comparison with SI and insular responses. Cereb. Cortex; 16:960-968 16177270 10.1093/cercor/bhj038

[b44] McCloskey D. I. 1978 Kinesthetic sensibility. Physiol. Rev.; 58:763-820 360251 10.1152/physrev.1978.58.4.763

[b45] McFarland N. R. Haber S. N. 2001 Organization of thalamostriatal terminals from the ventral motor nuclei in the macaque. J. Comp. Neurol.; 429:321-336 11116223 10.1002/1096-9861(20000108)429:2<321::aid-cne11>3.0.co;2-a

[b46] McIntyre A. K 1974 235-288 *in*In: Hunt C. C. (ed.). Central actions of impulses in muscle afferent fibres. Muscle Receptors Berlin Springer/

[b47] Mima T. Sadato N. Yazawa S. Hanakawa T. Fukuyama H. Yonekura Y 1999 Brain structures relating to active and passive finger movements in man. Brain; 122:1989-1997 10506099 10.1093/brain/122.10.1989

[b48] Mountcastle V. B 1974a 285-306 *in*In: Mountcastle V. B. (ed.). Sensory receptors and neural encoding: introduction to sensory processes. Medical physiology St Louis Mosby

[b49] Mountcastle V. B 1974b 307-347 *in*In: Mountcastle V. B. (ed.). Neural mechanisms in somesthesia. Medical physiology St Louis Mosby

[b50] Naito E. Roland P. R. Grefkes C. Choi H. J. Eickhoff S. Geyer S. 2005 Dominance of the right hemisphere and role of area 2 in human kinaesthesia. J. Neurophysiol.; 93:1020-1034 15385595 10.1152/jn.00637.2004

[b51] Niddam D. M. Yeh T.‐C. Wu Y.‐T. Lee P.‐L. Ho L.‐T. Arendt‐Nielsen L. 2002 Event‐related functional MRI study on central representation of acute muscle pain induced by electrical stimulation. Neuroimage; 17:1437-1450 12414283 10.1006/nimg.2002.1270

[b52] Ohye C. Shibazaki T. Hirai T. Wada H. Hirato M. Kawashima Y. 1989 Further physiological observations on the ventralis intermedius neurons in the human thalamus. J. Neurophysiol.; 61:488-500 2709095 10.1152/jn.1989.61.3.488

[b53] Ojemann G. A. Corina D. P. Corrigan N. Schoenfield‐McNeill J. Poliakov A. Zamora L. 2010 Neuronal correlates of functional magnetic resonance imaging in human temporal cortex. Brain; 133:46-59 19773355 10.1093/brain/awp227 PMC2801320

[b54] Ostrowsky K. Magnin M. Ryvlin P. Isnard J. Guenot M. Mauguière F. 2002 Repesentation of pain and somatic sensation in the human insula: a study of the responses to direct electrical cortical stimulation. Cereb. Cortex; 12:376-385 11884353 10.1093/cercor/12.4.376

[b55] Penny W. D. Holmes A. J. 2007 156-165 *in*In: Friston K. J. Ashburner J. T. Kiebel S. J. Nichols T. E. Penny W. D. (eds.). Chapter 12, Random effects analysis. Statistical parametric mapping London Academic Press

[b56] Proske U. 2006 Kinesthesia: the role of muscle receptors. Mus. Ner.; 34:545-558 10.1002/mus.20627 16897766

[b57] Proske U. Gandevia S. C. 2012 The proprioceptive senses: their roles in signalling body shape, body position and movement, and muscle force. Physiol. Rev.; 92:1651-1697 23073629 10.1152/physrev.00048.2011

[b58] Rothwell J. C. Traub M. M. Day B. L. Obeso J. A. Thomas P. K. Marsden C. D. 1982 Manual performance in a deafferented man. Brain; 105:515-542 6286035 10.1093/brain/105.3.515

[b59] Ruben J. Schwiemann J. Deuchert M. Meyer R. Krause T. Curio G. 2001 Somatotopic organization of human secondary somatosensory cortex. Cereb. Cortex; 11:463-473 11313298 10.1093/cercor/11.5.463

[b60] Schaechter J. D. Perdue K. 2008 Enhanced cortical activation in the contralesional hemisphere of chronic stroke patients in response to motor skill challenge. Cereb. Cortex; 18:638-647 17602141 10.1093/cercor/bhm096

[b61] Schmahmann J. D. Doyon J. McDonald D. Holmes C. Lavoie K. Hurwitz A. S. 1999 Three‐dimensional MRI atlas of the human cerebellum in proportional stereotaxic space. Neuroimage; 10:233-260 10458940 10.1006/nimg.1999.0459

[b62] Sengul G. Watson C. 2012 233-258 *in*In: Mai J. K. Paxinos G. (eds.). Spinal cord: connections. The Human Nervous System3rd edn. London Academic Press

[b63] Sherrington C. S. 1894 On the anatomical distribution of nerves of skeletal muscles; with remarks on recurrent fibres in the ventral spinal nerve root. J. Physiol. (Lond.); 17:211-258 10.1113/jphysiol.1894.sp000528 PMC1514567 16992213

[b64] Spiegel J. Tinera J. Gawehn J. Stoeter P. Treede R. D. 1999 Functional MRI of human primary somatosensory and motor cortex during median nerve stimulation. Clin. Neurophysiol.; 110:47-52 10348320 10.1016/s0168-5597(98)00043-4

[b65] Svensson P. Minoshima S. Beydoun A. Morrow T. J. Casey K. L. 1997 Cerebral processing of acute skin and muscle pain in humans. J. Neurophysiol.; 78:450-460 9242293 10.1152/jn.1997.78.1.450

[b66] Takanashi M. Abe K. Yanagihara T. Sakoda S. Tanaka H. Hirabuki N. 2003 A functional MRI study of somatotopic representation of somatosensory stimulation in the cerebellum. Neuroradiology; 45:149-152 12684716 10.1007/s00234-002-0935-3

[b67] Talairach J. T. Tournoux P. 1988 Coplanar stereotaxic atlas of the human brain. 3‐Dimensional proportional system: an approach to cerebral imaging Stuttgart Thieme Medical

[b68] Tempel L. W. Perlmutter J. S. 1990 Abnormal vibration‐induced cerebral blood flow responses in idiopathic dystonia. Brain; 113:691-707 2364264 10.1093/brain/113.3.691

[b69] Thach W. T. 1967 Somatosensory receptive fields of single units in cat cerebellar cortex. J. Neurophysiol.; 30:675-696 6035687 10.1152/jn.1967.30.4.675

[b70] Vitek J. L. Ashe J. DeLong M. R. Alexander G. E. 1994 Physiologic properties and somatotopic organization of the primate motor thalamus. J. Neurophysiol.; 71:1498-1513 8035231 10.1152/jn.1994.71.4.1498

[b71] Weiller C. Juptner M. Rijntjes M. Leonhardt G. Kiebel S. Muller S. 1996 Brain representation of active and passive movements. Neuroimage; 4:105-110 9345502 10.1006/nimg.1996.0034

[b72] Yousry T. A. Schmid U. D. Alkadhi H. Schmidt D. Peraud A. Buettner A. 1997 Localization of the motor hand area to a knob on the precentral gyrus. A new landmark. Brain; 120:141-157 9055804 10.1093/brain/120.1.141

